# Employment Precarity Strengthens the Relationships Between the Dark Triad and Professional Commitment

**DOI:** 10.3389/fpsyg.2021.673226

**Published:** 2021-07-20

**Authors:** Leah M. Kaufmann, Melissa A. Wheeler, Victor E. Sojo

**Affiliations:** ^1^School of Behavioural and Health Sciences, Faculty of Health Sciences, Australian Catholic University, Sydney, NSW, Australia; ^2^Department of Management and Marketing, Swinburne Business School, Swinburne University of Technology, Hawthorn, VIC, Australia; ^3^Department of Management and Marketing, Faculty of Business and Economics, The University of Melbourne, Parkville, VIC, Australia

**Keywords:** dark triad, professional commitment, organizational commitment, precarious employment, career interruption, part-time employment, casual employment

## Abstract

The Dark Triad traits of Machiavellianism, narcissism, and psychopathy have been found to negatively impact work behaviors including information sharing, reporting of unethical conduct, and mistreatment of colleagues and subordinates. However, research has found the Dark Triad can also be related to forms of organizational commitment which underpin positive work behaviors, including engaging in tasks and duties beyond those required (i.e., “going above and beyond”). Professional commitment is a broader form of commitment that has been found to be significantly related to organizational commitment, sharing antecedents, and having similar outcomes. Professional commitment, the affective, normative, and continuance commitment toward one's profession or occupation, has the benefit of applying to individuals employed by organizations as well as those working for themselves or between jobs. In this research, we explore relationships between professional commitment, using previous research on the relationship between the dark triad traits of Machiavellianism, psychopathy, and narcissism and organizational commitment, as a basis for predictions. We also explored two forms of precarious employment (career interruptions and part-time or casual work) as possible moderators of the dark triad-professional commitment relationship. Participants were 184 Australian professionals (52.2% men), a slight majority of whom had experienced a career interruption (69.6%) or a year or more of part-time or casual employment (70.7%). The results showed that psychopathy had a negative association with affective commitment, whereas Machiavellianism was positively related to normative commitment, and narcissism was positively related to normative and continuance commitment. Using regression analysis, we found that among individuals who have worked in part-time/casual employment longer, Machiavellianism and psychopathy had significantly stronger negative associations with affective commitment. In contrast, among individuals who have had a significant career interruption, Machiavellianism had significantly stronger positive association with continuance commitment. These findings help expand our understanding of both the dark triad and its contingent impact on workers' attachment to their profession.

## Introduction

The Dark Triad (DT; Paulhus and Williams, 2002) characteristics of Machiavellianism, narcissism, and psychopathy have been connected to negative consequences in the workplace, including being associated with counterproductive workplace behaviors (CWBs; O'Boyle et al., 2012), poor job performance (Le Breton et al., 2018), and low organizational citizenship (e.g., Becker and Dan O'Hair, 2007). Much of this research is underpinned by the reasoning that the pervasive socially indifferent (at best) to malevolent (at worst) motivations associated with DT traits lead to negative social behaviors in and consequences for workplaces. More recently, however, research has begun to explore beyond these basic relationships to understand the impact of contextual factors on DT and work behaviors (e.g., Webster and Smith, 2019). Findings from this research reveal that the DT interacts with factors such as opportunities to behave unethically (e.g., Harrison et al., 2018) or to avoid organizationally responsible action (e.g., Lyons et al., 2020) in predictably negative ways. Yet, DT can also enhance aspects of organizational citizenship (e.g., AL-Abrrow et al., 2020) and organizational commitment (Cesinger et al., 2018; Sahin and Ermis, 2020). The current research makes an important and unique contribution to this literature by exploring the impact of precarity at work. Precarity has been identified as a fundamental challenge to the concept of “decent work” (Blustein et al., 2016) and yet is an increasingly widespread (e.g., Johannessen, 2019) and consequential (e.g., Meuris and Leana, 2018) circumstance of contemporary professional life. We will specifically explore the impact of precarious work on the relationship between the DT and organizational commitment (e.g., Meyer and Allen, 1991).

### DT in the Workplace

The DT was first introduced as a related set of socially malevolent traits, some of which included self-promotional or aggrandizing features (Paulhus and Williams, 2002). As a result, the DT has often been conceptualized and used as a single or composite concept (e.g., Jonason et al., 2012; AL-Abrrow et al., 2020), although each characteristic in the triad has distinct features. Specifically, Machiavellianism is characterized by the manipulation and exploitation of others for personal gain, coldness, and selfishness (Christie and Geis, 1970; Szabó et al., 2018). Subclinical narcissism is characterized by entitlement, grandiosity, and a desire for dominance and superiority over others (Raskin and Hall, 1979). Finally, subclinical psychopathy encompasses thrill-seeking behavior, impulsivity, high anxiety, and a lack of empathy (Hare, 1985).

While DT characteristics have important consequences for the individuals themselves (e.g., Egan et al., 2014; Aghababaei and Błachnio, 2015), these characteristics are of significant interest in the workplace because of their important interpersonal, social, and professional outcomes. Specifically, DT traits undermine the development of strong collegiate relationships and the ability to collaborate in service of professional or even organizational goals (e.g., Szabó et al., 2018). In interactions with others in the workplace, Machiavellians aim to create positive impressions, especially when behaving like a team player can lead to the exchange of favors. These individuals typically use “soft tactics” such as compliments to achieve their own goals (Bereczkei et al., 2010; Jonason et al., 2012). In contrast, individuals scoring highly on subclinical psychopathy have little interest in others' perceptions of them; they tend to focus on short-term personal goals and willingly use “hard tactics” (e.g., bullying; Jonason et al., 2012). Finally, individuals scoring highly on subclinical narcissism can be unpredictable in their use of “soft” and “hard tactics” due their high self-perceived self-worth and belief that they are above the rules (Jonason et al., 2012), which is typically paired with a motivation to be admired (Bourdage et al., 2012). As a result, both Machiavellian and narcissistic individuals may have a slight or at least short-term advantage, being more agreeable and easier to work with than those who have psychopathic traits; although, when the pattern of self-serving priorities becomes apparent, co-workers can become irritated and feel alienated (e.g., DuBrin, 2012). Thus, as is easily apparent from these descriptions, individuals who score high on the DT are self-oriented and detrimentally driven to serve their own interests which can impact professional relationships, workplace harmony and collaboration, and the potential to achieve professional and organizational goals.

A social exchange account of the DT in the workplace puts the self-serving motive at the source of the failure to comply with norms (e.g., fairness, reciprocity). O'Boyle et al. (2012) posit that, while there are important distinctions between DT traits, “their basic strategy is one of apparent and covert exploitation of conspecifics” (p. 558) in service of own desires, although how this is achieved may differ across the three sub-categories. As a result, they reasoned that DT traits would be related to CWBs including interpersonal incivility and bullying, unethical conduct which can damage workplace culture, and lower job performance affecting organizational goals such as profitability. Based on a meta-analysis of 49 samples (*n* = 11,312), they found relationships between each of the DT traits and CWBs ranging from a moderate weighted correlation between narcissism and CWBs to a small but significant relationship between psychopathy and CWBs. Negative relationships were also observed between job performance, and Machiavellianism and psychopathy based on 143 samples (*n* = 19,836). No relationship was observed between job performance and narcissism in this meta-analysis, although Le Breton et al. (2018) suggest this evidence should be interpreted with caution due to the reliance on self-report measures, which likely reflect the tendency of narcissists to self-promote especially about their achievements.

Le Breton et al. (2018) conducted their own review of the impact of DT traits in the workplace, reviewing job performance, innovation and creativity, CWBs, job and work attitudes including job satisfaction, leadership, and organizational citizenship. Across these topics, they noted a trend for research identification of relationships between the DT and outcomes to be superseded in more recent research by identification of specific relationships between DT traits and outcomes. In addition, it was a feature of contemporary DT research to at least acknowledge the problems of self-report measures. For example, the self-promotional aspect of narcissism seemed to have led to the over-reporting of positive outcomes associated with this trait (e.g., strong relationships between self-reported but not objectively measured creativity and narcissism; Dahmen-Wassenberg et al., 2016). Finally, the authors emphatically called for future research to contextualize the effects of the DT on workplaces outcomes (e.g., O'Boyle et al., 2012) by identifying moderators and mediators that can enhance the real-world meaningfulness of these findings. One article receiving Le Breton et al. (2018) praise was conducted by Spain et al. (2016), who found that the DT traits typically reduce leadership effectiveness, can impact an organization beyond the direct leadership line, and can affect job performance and attitudes. Le Breton et al. (2018) noted that specific DT traits have differential impacts (e.g., for employee's well-being; Volmer et al., 2016) and differ in effectiveness as a result of features of the leader/subordinate pairings (e.g., Belschak et al., 2015; De Hoogh et al., 2015) and position in the organizational hierarchy (e.g., Wisse and Sleebos, 2016).

Of interest to the current research was Le Breton et al. (2018) identification of topics that were under-researched. For example, they highlighted organizational citizenship, defined inclusively as “a form of positive or prosocial workplace behaviors that are typically conceptualized as occurring outside one's focal employment role/job” (p. 395), to have important impacts but has yet to receive considerable attention. Another related topic in need of significant attention by researchers is organizational commitment. It is also interesting to note that one of the three findings Le Breton et al. (2018) cited as evidence of a negative relationship between the DT trait psychopathy and organizational citizenship was actually described by its authors as a study of psychopathy, social responsibility, and organizational commitment (Boddy et al., 2010). Organizational commitment, described as the individual's attachment to and identification with the organization (e.g., Mowday et al., 1979), has been found to be a strong predictor of organizational citizenship (e.g., Bakhshi et al., 2011), as well as to have important organizational outcomes including employee turnover (Jaros et al., 1993) and job performance (Riketta, 2002). Thus, in the current study we aimed to address this topic for further exploration of the important association between DT and professional commitment in the workplace.

### The DT and Professional Commitment

The literature on the DT and relationships with professional commitment, like research on DT relationships with organizational citizenship is relatively new and limited in scope. To our knowledge, previous research has almost exclusively explored the relationship between DT and organizational commitment (i.e., commitment to one's *organization*), but not professional commitment (i.e., commitment to one's *profession*). This literature thus far has also been somewhat inconsistent in both the measures and approaches used. For example, some studies have used the typical approach to studying individual differences, which involves asking participants to self-report or self-rate their tendencies and behaviors (e.g., Sahin and Ermis, 2020). However, citing the nature of the characteristics under investigation – both the inherent undesirability of the DT and the desirability of organizational commitment to those inclined to impression management (e.g., O'Boyle et al., 2012; Kowalski et al., 2018) – other studies have taken the less common approach of having participants rate others (e.g., managers) on the DT and report on their own organizational commitment. For example, Boddy et al. (2010) found that managers who were rated as high in psychopathy by employees were also rated as demonstrating little commitment to and recognition of employees compared to managers with lower levels of psychopathy. Using a similar approach, employee-rated psychopathic leadership (i.e., hostile, abusive, rude) was significantly related to lower endorsement of organizational values or acceptance of organizational norms (e.g., affective and normative commitment), and higher levels of commitment to remain with the organization by employees (e.g., Tepper, 2000). Similarly, Sanecka (2013) found employees who rated their managers as high in psychopathy reported significantly lower levels of normative and affective commitment, although no significant relationship was found with continuance commitment for this sample. These findings provide evidence that how employees perceive their managers' degree of psychopathy has important consequences for the organizational commitment of employees, regardless of how this outcome is assessed.

The only study to use self-reported measures and a single DT trait explored the relationships between workers' career commitment (i.e., self-focused), organizational commitment (i.e., to other workers, supervisors, and the organization), and Machiavellianism (Zettler et al., 2011). In a study of 154 German workers who had held roles with the same organization for 6 months or more, the authors test the reasoning that Machiavellians would show “a preference for taking advantage of opportunities in order to maximize own profits” (p. 22). From this, they hypothesized a positive relationship between Machiavellianism and (own) career commitment and negative relationships between Machiavellianism and other-benefitting organizational commitment. All predictions were supported.

Research examining relationships between self-reported DT and organizational commitment included Sahin and Ermis (2020) finding that, among Faculty of Sports Science academics in Turkey, all DT traits were significantly positively related to continuance commitment, but no other significant relationships were observed. Similarly, Cesinger et al. (2018) found significant positive relationships between narcissism, and continuance and normative commitment for French managers. Finally, Lyons et al. (2020) explored the relationships between DT traits and a single construct measure of organizational commitment and the potential effect of these relationships of the reporting of CWBs. They found Machiavellianism was negatively related to organizational commitment, while both Narcissism and psychopathy were positively related to organizational commitment of MTurk recruited workers. In addition, they found that high levels of organizational commitment reduced the reporting of CWBs by people who scored highly in narcissism and psychopathy. Taken together, these results provide evidence of a number of distinct and disparate relationships that could be affected by different measures and methods. The recency of the studies described above (e.g., post 2011) suggests that DT and career and organizational commitment are of growing interest and have the potential to contribute significantly to the understanding of the impact and experience of DT on work-related outcome.

To progress the aims of research on the DT and work-related commitment, research using established measures of these variables is needed both within diverse samples of workers, including those who are self-employed, and both across and within specific sectors. However, one challenge resulting from the aim of undertaking research with a diverse sample is the applicability of the concept of organizational commitment, specifically. For example, questions asking self-employed participants about their commitment to the organization may be difficult to compare to those of employed participants. For this reason, we assessed the broader yet related concepts of commitment to the profession (e.g., Meyer et al., 1993). Specifically, previous research found strong positive correlations between affective, normative, and continuance commitment to the organization and to the profession, as well as common antecedents and similar consequences (Meyer et al., 1993). Thus, by using a measure of professional commitment in place of organizational commitment, we afford greater inclusivity in our sample, while contributing to the current literature, and enhancing confidence in general relationships between the broad constructs. In addition, we will investigate the potential to identify contextual factors affecting when and where DT traits give rise to specific impacts and outcomes on professional commitment (e.g., Wisse and Sleebos, 2016). One contextual factor of interest is precarity.

### Employment Precarity as a Contextual Factor

Even preceding the impacts of the COVID-19 pandemic on global workplaces, disturbing trends have been observed toward increasingly insecure employment and rising numbers of precariously employed workers (e.g., Johannessen, 2019). The term precarious is specifically used in place of the narrower terms “insecure,” “informalized,” or “flexibilized” (e.g., Arnold and Bongiovi, 2013), as it acknowledges the inherent uncertainty of exploitative states of employment and potential for employment discrimination that include being underemployed (i.e., short term or reduced fraction contracts that are task based in place of broad and ongoing positions), underpaid (i.e., appointments that are not commensurate with skills or experience), extra-organizational (e.g., limited contract-based rather than appointed roles to bring in unique knowledge or skills), having low professional status (e.g., migrant workers, working mothers, and those on maternity, all positions characterized by a lack of power; Johannessen, 2019) or being at a disadvantage in the job market due to previous career interruptions (Evertsson et al., 2016).

To date, research on precarity has focused on constructs such as social movements, labor markets, discrimination, and policies and reform (e.g., Vosko et al., 2003; Kalleberg and Hewison, 2013; Sojo et al., 2016). Despite the acknowledgment that vulnerability both to, and as a result of precarity differs as a function of individuals' characteristics (e.g., gender, age, ethnicity, family responsibility, citizenship, e.g., Bacchetta et al., 2009), there is growing literature on the experiences and consequences of precarity for individuals (e.g., Lee et al., 2018), with evidence that precarity undermines work performance (e.g., Meuris and Leana, 2018).

To our knowledge, there is currently no research on the relationships between precarity and the DT or on how precarity can impact the DT- organizational or professional commitment relationship. However, we think there are good reasons for work precarity to play a role in the DT-professional commitment relationship. Precarity has been associated with lower organizational commitment, work engagement and well-being (Lee et al., 2018). In particular, work precarity appears associated with both lower sense of control (Vander Elst et al., 2014) and more personal sacrifices (e.g., not being able to buy groceries or clothing; Lozza et al., 2013), which are inherently stressful events that can impact the work attitudes and well-being of employees. Precarious work conditions would in themselves impair the fulfillment of basic needs for belonging, autonomy, and competence (Vander Elst et al., 2012). Such difficult situation can become a motivational force changing the way DT traits relate to professional commitment.

### The Current Study

The current study explored the relationship between the DT traits and professional commitment of Australian workers. This research was designed as an anonymous survey of workers using established measures of the DT (Jonason and Webster, 2010) and professional commitment (Meyer et al., 1993). We have used professional version of the dominant approach to organizational commitment which comprises the three elements of emotional attachment to or identifications with professional values and goals (i.e., affective commitment); a desire to remain in the profession because of the benefits or perceived lack of better alternative (i.e., continuance commitment); and an acceptance or obligation of service through one's profession (i.e., normative commitment: e.g., Meyer et al., 1993). Professional commitment was chosen because it has the benefit of being more inclusive of workers in a variety of situations (e.g., self-employed). Based on evidence that professional commitment is significantly correlated with organizational commitment, and has shared antecedents and similar consequences (e.g., Meyer et al., 1993), this construct assesses workers' identification with, feelings about, and obligation to belonging to their profession or occupation which can reasonably be expected to differentially relate to each of the DT traits as a result of the potential benefit to self, vs. others and the profession. We hypothesized that, based on Cesinger et al. (2018) findings, there would be significant positive relationships between narcissism, and continuance and normative commitment (Hypotheses 1A and 1B). Based on Zettler et al. (2011) findings, we hypothesized that Machiavellianism would be related to normative commitment, consistent with their reasoning that these individuals would endorse opportunities created by professional obligations as opportunities to personally benefit (e.g., reciprocity; Hypothesis 2). Finally, we hypothesized that psychopathy would have a significant negative relationship with affective commitment (Hypothesis 3), based on previous research that demonstrated this association between the related measure of other-reported ratings of psychopathy and affective commitment (e.g., Tepper, 2000; Boddy et al., 2010; Sanecka, 2013).

In addition to professional commitment, we asked participants to report on the current work (i.e., salary and whether they were in a management position) as well as a brief work history via a self-report resumé questionnaire. This questionnaire included items assessing experienced precarity such as the experience of career interruptions and part-time or casual employment. Previous research has typically found psychopathy is negatively related to objective markers of career success (e.g., salary, seniority; Ullrich et al., 2008), while Machiavellianism and narcissism have both been found to be related to a range of indicators of success (e.g., Ng et al., 2005; Spurk et al., 2016). However, there is no research on which to base specific predictions about the relationships between DT and precarity. We reason that psychopathy may, on the one hand, be linked to higher levels of precarity as a result of the previously demonstrated high levels of involuntary intraorganizational repositioning and termination (Spain et al., 2014). However, it is not clear that this would be specifically related to career interruption (e.g., family, retraining) or part-time and casual employment. In contrast, it can be expected that narcissism, which has been found to be related to achievement orientation (Judge and Bretz, 1992) and high self-efficacy beliefs (e.g., Sedikides et al., 2004), may be associated with undertaking a career interruption in service of professional or personal development with little concern about its impact. Narcissists seem unlikely to experience extended career interruptions, most of which occur in the service of others (e.g., family responsibilities or as a carer). For this reason, we predict that narcissism will be negatively related to career interruptions, and because of their tendency for professional success, that narcissism will also be negatively related to time in part-time and casual employment. As mentioned above, Machiavellians benefit from their ability to use soft manipulation tactics which are most effective within relationships and organizations. Consequently, Machiavellianism is likely to also be negatively related to career interruptions or to time in part-time and casual employment.

Given the predictions made about the relationship between DT traits and precarity, we do not have specific hypotheses about the impact of precarity on DT relationships and professional commitment. If relationships are observed between DT and professional commitment as predicted, the impact of precarity–as measured by career interruptions or time in part-time and casual employment–will be explored.

## Method

### Participants

Participants were 184 employed adult Australians recruited by students enrolled in an undergraduate psychology unit. The sample comprised 88 women (*M*_age_ = 37.94, *SD* = 12.91) and 96 men (*M*_age_ = 35.92, *SD* = 14.18). The majority of participants were born in Australia (86.4%), with the remaining participants born in Europe (6.0%), the South Pacific (2.7%), Asia (1.6%), and the Middle East, South America, and Africa (all 1.1%). Participants' responses were analyzed if they responded to >80% of items and included gender, age, and occupation details. A further 80 incomplete responses (30.3% of the 264 responses commenced) were omitted from analysis for failure to complete the designated 80%. This is consistent with previous research that recruited widely for an employed adult sample [e.g., 32.2% excluded by Zettler et al. (2011)].

### Measures

#### Demographics

Participants reported their gender, age, country of birth, and highest qualification.

#### Brief Resume

Participants reported on a variety of work experiences including years of employment, years of full-time employment, whether the current role involves managing people, yearly salary, experience of a career interruption, reason for career interruption (e.g., study, family responsibilities), and duration of career interruption.

#### Dark Triad

Participants self-rated items assessing Machiavellianism, psychopathy, and narcissism using the 12-item “Dirty Dozen” concise measure of the DT (Jonason and Webster, 2010). The items ask participants to endorse negative self-descriptions (i.e., “I tend to lack remorse”) on a 9-point Likert scale from 1 (*strongly disagree*) to 9 (*strongly agree*). DT trait subscales were scored by averaging the responses to relevant items. All scales demonstrated good reliability in this study (all α's > 0.83).

#### Professional Commitment

Participants completed the Meyer et al. (1993) Professional Commitment scale assessing affective commitment, continuance commitment, and normative commitment toward their “profession.” Each subscale comprised six items that participants responded to on a 7-point Likert scale from 1 (*strongly disagree*) to (*strongly agree*). After reverse scoring items describing low levels of commitment, each subscale was scored by averaging items. All scales demonstrated good reliability in this study (all α's > 0.82).

### Procedure

Participants were recruited by undergraduate psychology students consistent with the protocol used by Becker and Dan O'Hair (2007). This approach ensures that participants have no direct contact with any named researcher which, in addition to the anonymity of responses was intended to afford participants with the opportunity to be less concerned by impression management which was identified as an important issue when undertaking research on the DT. Participants were provided with an information letter and a link to an online survey. Participants indicated their informed consent, then completed the demographic and resume measures. Finally, participants completed the measures of the DT and commitment to profession, as well as measure not used as part of this study^1^ in randomized order. Participation took ~40 min.

### Data Analysis Approach

We first report bivariate correlations, means, and standard deviation for the study variables. To test our hypotheses, we then conducted a series of ordinary least squares multiple regression analysis with 10,000 bootstrap samples using PROCESS v 3.5.2 (Hayes, 2018). In these multiple regressions, the three types of professional commitments (i.e., affective, continuance, and normative) were used as the criteria, and the three dark traits (i.e., Machiavellianism, narcissism, and psychopathy) alternating as the main predictors, with the two markers of precarious employment conditions (i.e., having had a significant career interruption and years of part-time and casual employment) as alternating moderators. To account for the possibility of Family Wise Error Rate, we performed the Holm-Bonferroni correction method (Holm, 1979) for the set of regressions predicting each criterion.

We also controlled for participants' gender (as male or female, since we did not have any non-binary participants), age in years, whether they had completed tertiary education or not, and whether they were in managerial roles or not. We contemplated controlling for years of employment, yet the correlation between this variable and age was *r* = 0.97, *p* < 0.01 and with a similar pattern with all other variables in our model.

To evaluate the interactions between the dark traits and the markers of precarious employment, we also used a test of higher order unconditional interactions. When interactions were significant, we estimated the conditional effects of the dark traits at levels of the precarious employment variables (i.e., significant career interruption: yes = 1, no = 0; years of part-time and casual employment: +/- 1 standard deviation from the mean and Johnson-Neyman intervals) and produced interaction graphs.

## Results

### Correlations

As shown in Table 1, in our sample, affective commitment was not significantly correlated with either continuance or normative commitment. However, continuance and normative commitment had a significant positive medium-sized correlation. Years in part-time and casual employment was not significantly related to any of the indicators of professional commitment. However, having experienced a career interruption had a significant positive but small correlation with affective commitment.

**Table 1:** Descriptive statistics and correlations for study variables.

	***M***	**SD**	**1**	**2**	**3**	**4**	**5**	**6**	**7**	**8**	**9**	**10**	**11**	**12**	**13**
1. Gender	0.48	0.50													
2. Age	36.89	13.59	0.08												
3. Education	0.55	0.50	0.01	0.12											
4. Years employed	19.59	13.34	0.05	0.97^**^	0.10										
5.Years PTCE	5.03	6.28	0.45^**^	0.25^**^	0.01	0.31^**^									
6. Manager	0.43	0.50	−0.19^**^	0.10	0.20^**^	0.11	−0.17^*^								
7. Career interruption	0.70	0.46	0.14	0.24^**^	−0.04	0.24^**^	0.22^**^	0.05							
8. Salary (Log)	10.94	0.49	−0.27^**^	0.42^**^	0.30^**^	0.40^**^	−0.06	0.26^**^	0.12						
9. Machiavelli-anism	4.06	1.94	−0.14	−0.24^**^	−0.05	−0.21^**^	−0.19^**^	0.04	−0.21^**^	−0.08					
10. Psychopathy	3.17	1.71	−0.30^**^	−0.18^*^	−0.06	−0.14^*^	−0.19^**^	0.10	−0.25^**^	<0.01	0.61^**^				
11. Narcissism	4.74	1.80	−0.16^*^	−0.17^*^	0.01	−0.19^*^	−0.15^*^	−0.05	−0.16^*^	−0.04	0.57^**^	0.45^**^			
12. Affective commit.	5.32	1.12	0.01	0.17^*^	0.08	0.17^*^	0.09	0.06	0.15^*^	0.20^**^	−0.06	−0.25^**^	−0.03		
13. Continuance commit.	4.14	1.44	−0.16^*^	0.28^**^	0.18^*^	0.25^**^	0.01	0.09	−0.03	0.23^**^	0.06	0.06	0.20^**^	−0.02	
14. Normative commit.	3.65	1.32	−0.07	−0.01	0.06	−0.01	−0.06	0.13	−0.03	0.07	0.20^**^	0.14	0.23^**^	0.05	0.41^**^

*Career interruption: 0 = no, 1 = yes. Gender: 0 = man, 1 = woman. Education: 0 = high school or lower, 1 = tertiary education. Manager: 0 = no, 1 = yes. Years PTCE = years of part-time and casual employment. N = 182–184.*

* *p < 0.05,*

** *p < 0.01*.

Consistent with Hypothesis 3, psychopathy had a significant negative correlation with affective commitment, that is, participants who reported psychopathic traits were less likely to have an emotional attachment with their professions. Also, consistent with Hypotheses 2, Machiavellianism had a significant positive small association with normative commitment, so that participants who reported manipulative tendencies were more likely to want to stay in their current occupations out of a sense of reciprocity. Similarly, consistent with Hypotheses 1A and 1B, narcissism had significant positive small correlations with both continuance and normative commitment. These results indicate that participants who are overly concerned with themselves are more likely to want to stay in their current profession. However, it seems likely that this is because they may not have better job prospects and because of a sense of reciprocity, consistent with the transactional nature of their attachment to their profession. We also observed that years in part-time and casual employment as well as having had important career interruptions had significant, small and negative associations with all the DT traits.

### Associations Between Dark Triad, Precarity, and Affective Commitment

The multiple regression analyses exploring further the association between the DT, precarious employment, and affective commitment are reported in Table 2. Psychopathy remained a significant negative predictor of affective commitment in the multivariate regressions (see Table 2, Panels A and B).

**Table 2:** Multiple regression coefficients and model estimates predicting affective commitment.

	**Affective commitment**								
	**Machiavellianism**			**Narcissism**			**Psychopathy**		
	**Coeff**.	**SE**	***p***	**Coeff**.	**SE**	***p***	**Coeff**.	**SE**	***p***
**Panel A**									
Constant	4.69	0.28	<0.01	4.67	0.27	<0.01	4.91	0.27	<0.01
Dark trait	−0.01	0.08	0.90	0.00	0.09	0.98	**−0.19**	**0.08**	**0.03**
Career interruption	0.31	0.19	0.12	0.31	0.19	0.11	0.20	0.19	0.31
DT × CI	0.00	0.10	0.99	0.01	0.11	0.90	0.04	0.10	0.70
Gender	−0.07	0.17	0.68	−0.06	0.17	0.72	−0.20	0.17	0.25
Age	0.01	0.01	0.13	0.01	0.01	0.11	0.01	0.01	0.21
Education	0.16	0.17	0.36	0.15	0.17	0.37	0.12	0.17	0.48
Manager	0.04	0.18	0.80	0.04	0.17	0.80	0.09	0.17	0.61
Model summary	*R^2^* = 0.05			*R^2^* = 0.05			*R^2^* = 0.10	
	*F*_(7, 174)_ = 1.27, *p* = 0.27			*F*_(7, 174)_ = 1.27, *p* = 0.27			*F*_(7, 174)_ = 2.75, *p* = 0.01		
**Panel B**									
Constant	4.81	0.28	<0.01	4.79	0.28	<0.01	4.99	0.27	<0.01
Dark trait	−0.03	0.04	0.48	0.00	0.05	0.92	−0.19	0.05	<0.01
PTCE	−0.01	0.02	0.76	0.01	0.02	0.58	0.00	0.02	0.94
DT × PTCE	**−0.02**	**0.01**	** <0.01**	−0.01	0.01	0.10	−0.02	0.01	0.02
Gender	−0.11	0.18	0.56	−0.10	0.19	0.59	−0.25	0.18	0.17
Age	0.01	0.01	0.07	0.01	0.01	0.06	0.01	0.01	0.21
Education	0.13	0.17	0.44	0.15	0.17	0.38	0.15	0.16	0.36
Manager	0.06	0.17	0.72	0.11	0.18	0.52	0.13	0.17	0.44
Model summary	*R*^2^ = 0.08		*R^2^* = 0.05		*R^2^* = 0.13			
	*F*_(7, 174)_ = 2.21, *p* = 0.04			*F*_(7, 174)_ = 1.36, *p* = 0.23			*F*_(7, 174)_ = 3.58, *p* <0.01		

*DT, Dark traits; CI, Career interruption. 0 = no, 1 = yes. Gender: 0 = man, 1 = woman. Education: 0 = high school or lower, 1 = tertiary education. Manager: 0 = no, 1 = yes. PTCE = years of part-time and casual employment. N = 182. Bold indicates interaction coefficients are statistically significant at 0.05 after Holm-Bonferroni corrections*.

We did not observe a significant interaction between the DT traits and career interruptions in their impact on affective commitment (see Table 2, Panel A). However, we found a significant interaction between Machiavellianism and years in part-time and casual employment (*R*^2^ change = 0.04, *F*_(1, 174)_ = 8.39, *p* < 0.01), and an interaction between psychopathy and years in part-time and casual employment (*R*^2^ change = 0.03, *F*_(1, 174)_ = 6.08, *p* = 0.02 marginally significant after applying the Holm-Bonferroni correction), in their impact on affective commitment.

**Figure 1:**
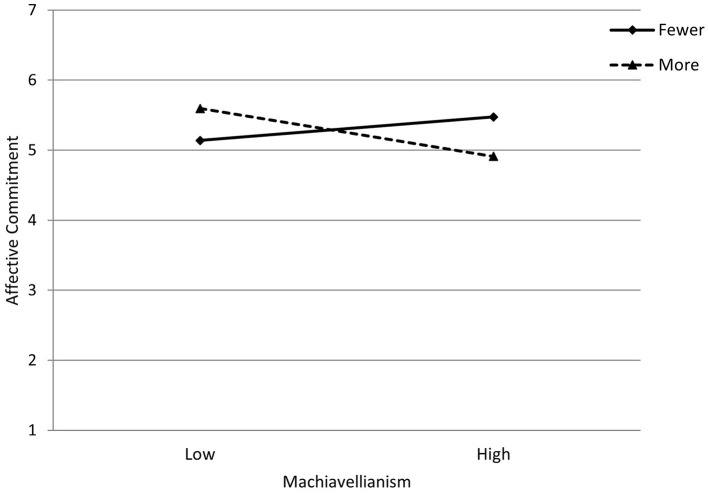
Interaction between Machiavellianism and years of part-time and casual employment in their impact on affective commitment. Lines represent years of part-time and casual work at −1SD = *Fewer* and +1SD = *More* from the mean.

As can be seen in Figure 1, for participants with fewer years of part-time and causal work (minus 1 SD from the mean) the association between Machiavellianism and affective commitment was positive but not statistically significant (coefficient = 0.09, *SE* = 0.06 [95% CI −0.03, 0.20]). However, for participants with more years of part-time and causal work (plus 1 SD from the mean) the association between Machiavellianism and affective commitment was negative and statistically significant (coefficient = −0.18, *SE* = 0.07 [95% CI −0.31, −0.04]).

To substantiate these results, we also calculated Johnson-Neyman intervals for this interaction. The results indicated that when participants had nine years of part-time and casual employment, Machiavellianism and affective commitment started having a significant negative association (coefficient = −0.12, SE = 0.06 [95% CI −0.23, −0.01]). As years of part-time and casual employment increases, the relationship between Machiavellianism and affective commitment becomes more negative, with the highest number of years (30 years) having an associated coefficient = −0.61, SE = 0.21 (95% CI −1.02, −0.20).

**Figure 2:**
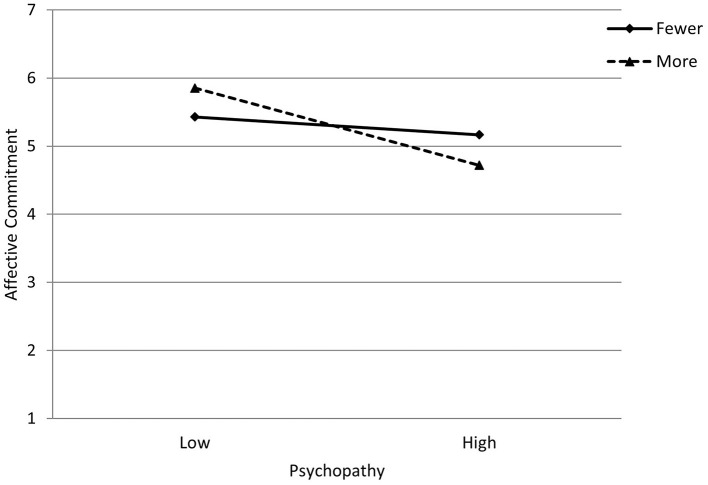
Interaction between psychopathy and years of part-time and casual employment in their impact on affective commitment. Lines represent years of part-time and casual work at −1SD = *Fewer* and +1SD = *More* from the mean.

A similar pattern was observed for participants with fewer years of part-time and causal work (minus 1 SD from the mean), among them the association between psychopathy and affective commitment was negative but not statistically significant (coefficient = −0.08, *SE* = 0.06 [95% CI −0.20, 0.05]). However, for participants with more years of part-time and causal work (plus 1 SD from the mean), the association between psychopathy and affective commitment was negative and statistically significant (coefficient = −0.34, *SE* = 0.08 [95% CI −0.50, −0.17]). Figure 2 illustrates this interaction, indicating that as years of working part-time and casual jobs increase, the relationship between psychopathy and affective commitment becomes more negative. The Johnson-Neyman intervals indicated that when participants had one and a half years of part-time and casual employment, psychopathy, and affective commitment started having a significant negative association (coefficient = −11, SE = 0.05 [95% CI −0.22, −0.01]. As years of part-time and casual employment increases, the relationship between psychopathy and affective commitment becomes more negative, with the highest number of years (30 years) having an associated coefficient = −0.76, SE = 0.24 (95% CI −1.23, −0.28). Nevertheless, this interaction should be interpreted with caution given it is only marginally significant.

### Association Between Dark Triad, Precarity, and Continuance Commitment

The multiple regression analyses exploring further the association between the DT, precarity, and continuance commitment are reported in Table 3. We found a significant interaction between Machiavellianism and career interruptions (*R*^2^ change = 0.04, *F*_(1, 174)_ = 8.23, *p* < 01) in their impact on continuance commitment (see Table 3, Panel A).

**Table 3:** Multiple regression coefficients and model estimates predicting continuance commitment.

	**Continuance commitment**								
	**Machiavellianism**			**Narcissism**			**Psychopathy**		
	**Coeff**.	**SE**	***p***	**Coeff**.	**SE**	***p***	**Coeff**.	**SE**	***p***
**Panel A**									
Constant	3.08	0.33	<0.01	3.00	0.33	<0.01	3.14	0.34	<0.01
Dark trait	−0.15	0.10	0.13	0.05	0.11	0.66	−0.10	0.11	0.37
Career interruption	−0.27	0.23	0.25	−0.16	0.23	0.49	−0.23	0.24	0.33
DT × CI	**0.33**	**0.12**	** <0.01**	0.18	0.13	0.16	0.21	0.13	0.10
Gender	−0.36	0.21	0.09	−0.38	0.21	0.07	−0.42	0.22	0.05
Age	0.03	0.01	<0.01	0.03	0.01	<0.01	0.03	0.01	<0.01
Education	0.38	0.21	0.07	0.37	0.20	0.07	0.37	0.21	0.08
Manager	0.06	0.21	0.77	0.05	0.21	0.81	0.03	0.22	0.88
Model summary	*R^2^* = 0.18			*R^2^* = 0.19			*R^2^* = 0.14	
	*F*_(7, 174)_ = 5.30, *p* <0.01			*F*_(7, 174)_ = 5.67, *p* <0.01			*F*_(7, 174)_ = 4.09, *p* <0.01		
**Panel B**									
Constant	3.02	0.35	<0.01	2.99	0.34	<0.01	3.11	0.35	<0.01
Dark trait	0.08	0.06	0.14	0.19	0.06	<0.01	0.05	0.07	0.44
PTCE	0.00	0.02	0.90	0.01	0.02	0.53	0.01	0.02	0.64
DT × PTCE	−0.01	0.01	0.30	0.00	0.01	0.89	0.00	0.01	0.76
Gender	−0.52	0.23	0.02	−0.45	0.22	0.04	−0.50	0.24	0.04
Age	0.03	0.01	<0.01	0.03	0.01	<0.01	0.03	0.01	<0.01
Education	0.43	0.21	0.04	0.40	0.20	0.07	0.43	0.21	0.04
Manager	−0.02	0.21	0.95	0.06	0.21	0.79	−0.01	0.22	0.97
Model summary	*R^2^* = 0.14		*R^2^* = 0.18		*R^2^* = 0.13			
	*F*_(7, 174)_ = 4.12, *p* <0.01			*F*_(7, 174)_ = 5.36, *p* <0.01			*F*_(7, 174)_ = 3.61, *p* <0.01		

*DT, Dark traits; CI, Career interruption. 0 = no, 1 = yes. Gender: 0 = man, 1 = woman. Education: 0 = high school or lower, 1 = tertiary education. Manager: 0 = no, 1 = yes. PTCE = years of part-time and casual employment. N = 182. Bold indicates interaction coefficients are statistically significant at 0.05 after Holm-Bonferroni corrections*.

**Figure 3:**
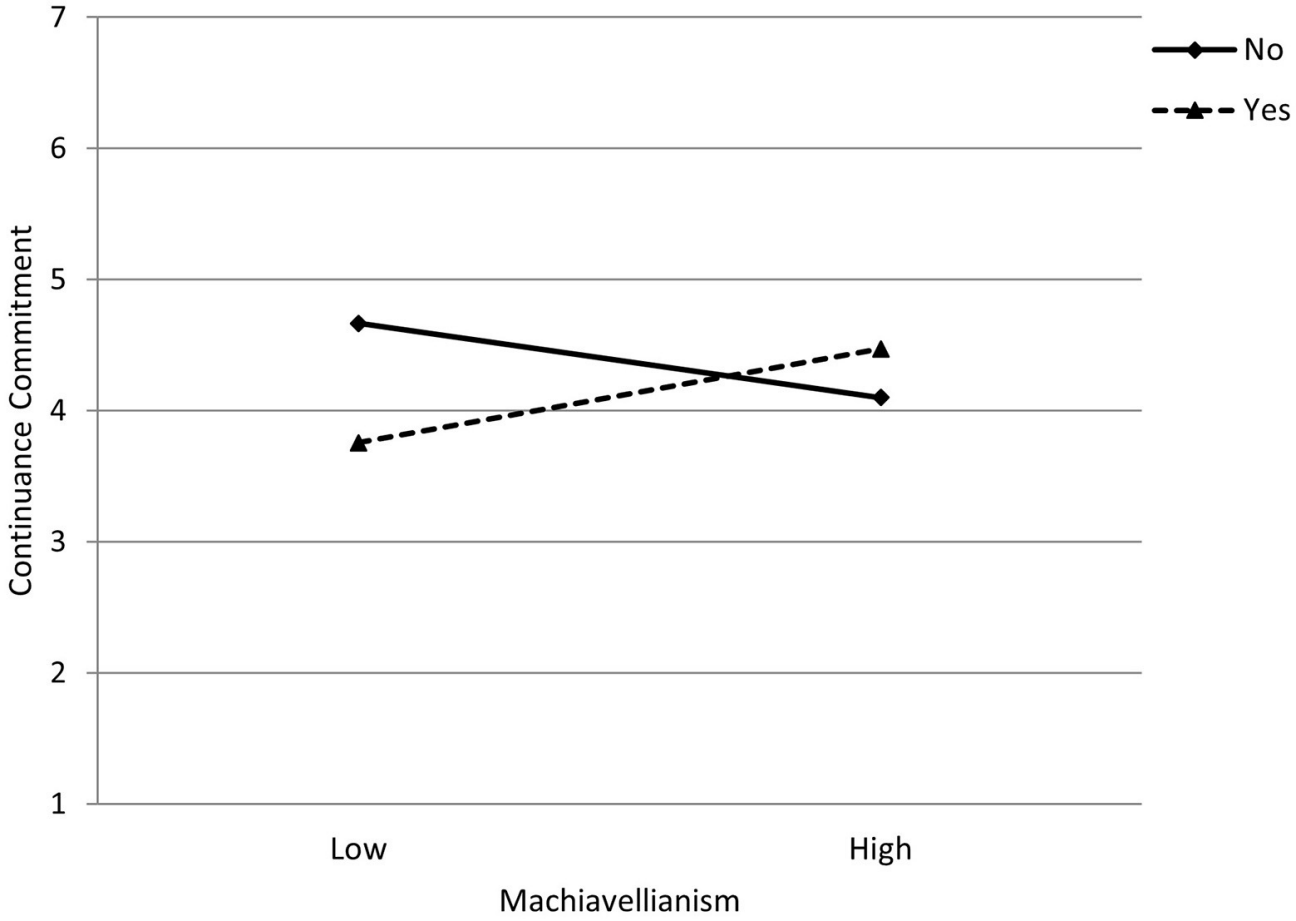
Interaction between Machiavellianism and career interruption in their impact on continuance commitment. Lines represent whether the participant did or did not report a significant career interruption.

For participants who have never had a significant career interruption, the association between Machiavellianism and continuance commitment was negative but not statistically significant (coefficient = −0.15, *SE* = 0.10 [95% CI −0.33, 0.04]). However, among participants who have had a significant career interruption, the association between Machiavellianism and continuance commitment was positive and statistically significant (coefficient = 0.19, *SE* = 0.07 [95% CI 0.06, 0.31]). Figure 3 illustrates this interaction, indicating that among participants with significant career interruptions, those who describe themselves as being more manipulative are more likely to be attached to their profession because of not having better employment prospects.

We did not observe significant interactions between the dark traits and years of part-time and casual employment in their impact on continuance commitment (see Table 3, Panel B).

### Association Between Dark Triad, Precarity and Normative Commitment

We did not observe significant interactions between the dark traits and career interruptions or years of part-time and casual employment in their impact on normative commitment (see Table 4, Panels A and B).

**Table 4:** Multiple regression coefficients and model estimates predicting normative commitment.

	**Normative commitment**								
	**Machiavellianism**	**Narcissism**	**Psychopathy**
	**Coeff**.	**SE**	***p***	**Coeff**.	**SE**	***p***	**Coeff**.	**SE**	***p***
**Panel A**									
Constant	3.42	0.33	<0.01	3.46	0.32	<0.01	3.52	0.33	<0.01
Dark trait	0.07	0.09	0.47	0.08	0.11	0.45	0.03	0.10	0.81
Career interruption	0.01	0.23	0.95	0.00	0.22	0.99	−0.01	0.23	0.96
DT × CI	0.10	0.11	0.37	0.12	0.13	0.33	0.10	0.13	0.43
Gender	−0.02	0.20	0.91	−0.01	0.20	0.97	−0.02	0.21	0.93
Age	<0.01	0.01	0.85	0.00	0.01	0.96	<0.01	0.01	0.93
Education	0.12	0.20	0.55	0.08	0.20	0.70	0.11	0.21	0.60
Manager	0.29	0.21	0.17	0.33	0.20	0.11	0.29	0.21	0.12
	*R^2^* = 0.06			*R^2^* = 0.08			*R^2^* = 0.03	
	*F*_(7, 174)_ = 1.62, *p* = 0.13			*F*_(7, 174)_ = 2.08, *p* = 0.05			*F*_(7, 174)_ = 0.87, *p* = 0.54		
**Panel B**									
Constant	3.44	0.33	<0.01	3.47	0.33	<0.01	3.53	0.34	<0.01
Dark trait	**0.14**	**0.05**	**<0.01**	**0.17**	**0.01**	**<0.01**	0.10	0.06	0.13
PTCE	<0.01	0.02	0.88	0.00	0.02	0.93	<0.01	0.02	0.95
DT × PTCE	<0.01	0.01	0.71	0.01	0.01	0.40	0.01	0.01	0.43
Gender	−0.05	0.22	0.84	<0.01	0.22	1.00	−0.01	0.23	0.97
Age	<0.01	0.01	0.88	<0.01	0.01	0.99	<0.01	0.01	0.90
Education	0.13	0.20	0.52	0.08	0.20	0.70	0.11	0.20	0.59
Manager	0.27	0.21	0.19	0.32	0.21	0.13	0.26	0.21	0.22
Model summary	*R^2^* = 0.06			*R^2^* = 0.08			*R^2^* = 0.03		
	*F*_(7, 174)_ = 1.50, *p* = 0.17			*F*_(7, 174)_ = 2.04, *p* = 0.05			*F*_(7, 174)_ = 0.87, *p* = 0.53		

*DT, Dark traits; CI, Career interruption. 0 = no, 1 = yes. Gender: 0 = man, 1 = woman. Education: 0 = high school or lower, 1 = tertiary education. Manager: 0 = no, 1 = yes. PTCE = years of part-time and casual employment. N = 182. Bold indicates interaction coefficients are statistically significant at 0.05 after Holm-Bonferroni corrections*.

## Discussion

In the current study, we explored the relationship between the DT (Machiavellianism, psychopathy, and narcissism) and the under-researched workplace variable of professional commitment (affective, normative, and continuance). We found support for our three hypotheses. First, psychopathy, a trait characterized by low emotional attachment and concern, was negatively related to affective commitment, showing a lack of attachment to these individuals' profession. Machiavellianism, on the other hand, was positively related to normative commitment. People who score high on Machiavellianism typically demonstrate manipulative tendencies which may increase their sense of loyalty as a means to secure the personal returns on their investments in the workplace and with colleagues both in and beyond a specific workplace. As a result, they foster interpersonal relationships to achieve personal goals, illustrating a transactional approach to professional commitment.

The third DT trait, narcissism, was found to be positively related to both continuance and normative commitment. Those who are overly focused on themselves and how they are perceived are more likely to stay in their profession because they perpetually seek recognition for what they believe are their typically above average contributions and achievements and, unless they envision better prospects for themselves elsewhere, this binds them to their current profession. Similarly, narcissists are more likely to show loyalty to their professions because of a sense of reciprocity for what they have gained from the profession and the status they might get from valuing their jobs, highlighting the self-serving way narcissists commit to their profession. While narcissist might endorse a sense of loyalty with their professions, such loyalty can change when they find something better, as it is indicated by their higher continuance commitment, similar patterns have been observed among narcissists with brand loyalty (Lambert and Desmond, 2013).

Relationships between DT traits and precarity revealed, consistent with predictions that Machiavellianism and narcissism were significantly negatively related to career interruptions and to years in part-time and casual employment. The finding for psychopathy was inconsistent with the prediction that these individuals may experience more years in part-time and casual employment but was consistent with the findings for DT traits. This finding suggests that psychopathy may be related to high levels of intraorganizational movement and job loss (e.g., Spain et al., 2014), but does not seem to mean these individuals spend time out of work (e.g., career interruption) or in precarious (i.e., part-time or casual) employment. It may be that subclinical psychopathy is not associated with this disadvantage.

Analyses exploring the impact of precarious employment (i.e., career interruption or years in casual or part-time employment) on the relationship between DT and professional commitment revealed that, for those who experienced precarious employment as casuals or part-timers, there was a significant negative association between both Machiavellian and psychopathy and affective commitment. This relationship that was not present for Machiavellians in continuous secure employment. Interestingly, we also observed that the experience of a career interruption, another factor increasing the precarity of employment, strengthened the existing positive relationship between Machiavellianism and continuance commitment. Precarity was found to have no significant impacts on the relationship between narcissism and professional commitment. Taken together, these findings suggest that precarity strengthened the relationships between DT and professional commitment with the effects of increasing the emotional detachment with their professions of those high in psychopathy and Machiavellianism yet enhancing the tendency of the latter to be more transactional in their engagement with their profession, in particular by showing commitment because they do not have better prospects in other occupations.

The current findings contribute to the limited research on the relationship between DT and commitment in relation to work, by providing evidence of relationships between DT and professional commitment within a diverse cross-sectional sample of workers and identifying some impact of precarity on these relationships. We observed that, consistent with previous research, participants' self-rated narcissism was related to continuance and normative commitment, which is consistent with Cesinger et al. (2018). The negative relationship between worker's psychopathy and affective commitment is similar to Sanecka (2013) results; however, she found an interpersonal effect, with managers' psychopathy resulting in decreased employee commitment when both were reported by the employee. Finally, our finding that Machiavellians demonstrated high levels normative commitment to the profession seems consistent with Zettler et al. (2011) findings that Machiavellians endorsed items such as “my career has a great deal of personal meaning for me” (p. 24) which they labeled “career commitment,” but not scale reflecting attachment to the organization or other employees.

### Contributions

The current findings were consistent with previous research on DT and organizational commitment even though we used the related but more inclusive construct of professional commitment. As a result, we conclude that the effect of DT on professional commitment for those scoring highly on narcissism and Machiavellianism has some positive consequences for workplaces, which contrast the typically reported negative effects of these traits in and for workplaces (O'Boyle et al., 2012; Le Breton et al., 2018). For example, normative commitment is typically associated with positive discretionary workplace behaviors via the obligation to reciprocate perceived support from their organization. However, it seems likely that narcissistic and Machiavellian employees endorse the norm of reciprocity because it is a bidirectional obligation. As a result, normative commitment may be viewed as a mechanism to personal recognition, interpersonal control, or future opportunities which may have ambivalent consequences. This conclusion highlights a key contribution of research on DT and professional commitment traits that can only be achieved when these traits and factors are measured and examined using the distinct subscales. Such observations are also enhanced by the recruitment of a diverse sample (e.g., age, gender, range of employment) as was achieved in the current research.

The current research also makes a unique contribution to the extant literature, being the first study to consider these factors in conjunction with precarity. As we noted, precarious employment is an increasingly important factor to consider in workplace research given that it is now commonplace and continues to grow as an organizational strategy (e.g., Alberti et al., 2018) and as a pressure on workers (e.g., Lewchuk et al., 2016). Interestingly, we observed significant negative relationships between DT and part-time and casual employment, and career interruption indicating that those with higher levels of DT traits were less likely to report they had experienced precarity. Moreover, for those high in Machiavellianism and psychopathy, precarity appeared to exacerbate their effects on some forms of professional commitment.

### Limitations and Future Directions

The current research is limited to the discussion of associations between the factors of interest due to the use of a cross-sectional design. As a result, we are unable to draw conclusions about the direction of causation between these constructs. For example, we are unable to conclude that a period of career interruption increases normative commitment among Machiavellians, although such an occurrence seems logical due to the potential for precarity to reduce desirable alternatives, consequently bolstering the value of the current employer to transactionally oriented workers. The current findings do, however, provide a justification for future research that could address this limitation using a more resource-costly and robust longitudinal research design. Using such an approach would also permit consideration of current vs. previous experiences of precarity, changes in experiences of precarity, and the consequence of these on career trajectories and professional commitment.

A further limitation of the current research, like that of all research undertaken on DT, is that individuals high on these traits are differentially prone to impression management which can be problematic for measures that rely on self-report. Nonetheless, like other researchers we attempted to mitigate this motivation by using an established self-report measure with anonymous participation to limit the potential value of esteem (e.g., Sahin and Ermis, 2020) and arms-length recruiting practices [i.e., students rather than the researchers provided potential participants with the recruitment information; e.g., Becker and Dan O'Hair (2007)]. In addition, the DT measure was only one measure among several others, and was not labeled, although this would in no way obscure the inherent undesirability of the behaviors described in the questions (e.g., “I tend to lack remorse”; Jonason and Webster, 2010). Support for the effectiveness of this approach to limit desirable responding is provided by the finding that a full range of scores were observed within the current sample.

A final limitation of the current research was the use of the professional commitment rather than the more common organizational commitment, which meant that no direct comparison or replications were undertaken. As already noted, we used the broader concept which may have introduced greater variability in participants [e.g., we foresee concept of professional commitment may have more impact for professional occupations than for those in service occupations; for further discussion, see Meyer et al. (1993)]. Nonetheless, the current findings were highly consistent with previous research on DT and organizational commitment (e.g., Boddy et al., 2010; Zettler et al., 2011; Cesinger et al., 2018), and we recommend that future research that includes participants who are self-employed should consider using the variable of professional over organizational commitment.

## Conclusion

The current research presents evidence for the complex and ambivalent effects of the DT on workers and in workplaces via the concept of professional commitment. Moreover, we highlighted the impact of precarity as an increasingly common real-world workplace factor with the potential to exacerbate the impacts of DT in workplaces through the concept of commitment. In doing so, we have brought together the limited and disparate findings to progress the aims of research on the DT and professional commitment. We proposed that future research using established measures of DT and professional commitment variables, alongside objective measures of precarity, is needed both within diverse samples of workers and within specific sectors. Furthermore, this literature will benefit from research which permits examination of the consequential effect of precarity, via multiple observation or longitudinal methods, which will begin to highlight the pervasiveness of this factor and its long-term interactive effects for DT workers and their workplaces.

## Data Availability Statement

The datasets generated for this study can be found in online repositories. The names of the repository/repositories and accession number(s) can be found at: https://osf.io/egjrh. The datasets analyzed for this study can be found in the Open Science Framework repository as: LK, MW, and VS (2021, February 26). Employment Precarity Moderates the Relationships Between the Dark Triad and Professional Commitment.

## Ethics Statement

The studies involving human participants were reviewed and approved by Australian Catholic University Human Research Ethics Committee. Written informed consent for participation was not required for this study in accordance with the national legislation and the institutional requirements.

## Author Contributions

All authors listed have made a substantial, direct and intellectual contribution to the work, and approved it for publication.

## Conflict of Interest

The authors declare that the research was conducted in the absence of any commercial or financial relationships that could be construed as a potential conflict of interest.

## Footnotes

^1^A complete list of variables is provided with the data which is available from the Open Science Framework https://osf.io/egjrh.

## Abbreviations

CWB: Counterproductive workplace behaviors
DT: Dark triad
PTCE: Part-time or casual employment.
